# Acoustic change complex in background noise: phoneme level and timing effects

**DOI:** 10.14814/phy2.13464

**Published:** 2017-10-19

**Authors:** Curtis J. Billings, Leslie D. Grush, Nashrah Maamor

**Affiliations:** ^1^ National Center for Rehabilitative Auditory Research Veterans Affairs Portland Health Care System Portland Oregon; ^2^ Department of Otolaryngology Oregon Health & Science University Portland Oregon; ^3^ Audiology Program School of Rehabilitation Sciences Faculty of Health Sciences The National University of Malaysia Kuala Lumpur Malaysia

**Keywords:** Acoustic change complex, cortical auditory evoked potentials, neural refractory period, signal‐to‐noise ratio, speech stimuli

## Abstract

The effects of background noise on speech‐evoked cortical auditory evoked potentials (CAEPs) can provide insight into the physiology of the auditory system. The purpose of this study was to determine background noise effects on neural coding of different phonemes within a syllable. CAEPs were recorded from 15 young normal‐hearing adults in response to speech signals /s/, /ɑ/, and /sɑ/. Signals were presented at varying signal‐to‐noise ratios (SNRs). The effects of SNR and context (in isolation or within syllable) were analyzed for both phonemes. For all three stimuli, latencies generally decreased and amplitudes generally increased as SNR improved, and context effects were not present; however, the amplitude of the /ɑ/ response was the exception, showing no SNR effect and a significant context effect. Differential coding of /s/ and /ɑ/ likely result from level and timing differences. Neural refractoriness may result in the lack of a robust SNR effect on amplitude in the syllable context. The stable amplitude across SNRs in response to the vowel in /sɑ/ suggests the combined effects of (1) acoustic characteristics of the syllable and noise at poor SNRs and (2) refractory effects resulting from phoneme timing at good SNRs. Results provide insights into the coding of multiple‐onset speech syllables in varying levels of background noise and, together with behavioral measures, may help to improve our understanding of speech‐perception‐in‐noise difficulties.

## Introduction

Difficulty understanding speech in the presence of background noise is a commonly reported problem. This perceptual difficulty becomes increasingly severe as competing background noise levels increase (i.e., signal‐to‐noise ratio (SNR) decreases). As with speech recognition performance, neural responses are typically weakened when increasing levels of noise are present. In fact, for suprathreshold signals presented in background noise, SNR has been shown to override the effects of absolute signal level such that typical signal level effects are not found (Billings et al. 2015), which is something that also occurs perceptually (Hawkins and Stevens 1950). It is not surprising, then, that strong correlations exist between cortical auditory evoked potentials (CAEPs) and speech perception‐in‐noise measures (Billings et al. 2015).

There is growing interest in the use of more complex multiple‐onset speech stimuli (rather than single‐onset tones or speech sounds) to evoke CAEPs that may be more representative of everyday speech. The use of more complex speech stimuli may improve our understanding of the processing of real‐world speech. In a widely cited early demonstration of this effect, Ostroff et al. (1998) showed multiple N1‐P2 responses to the syllable /sei/: one response to the onset of the fricative /s/ and another to the onset of the vowel. The second N1‐P2 response has been referred to as the acoustic change complex (ACC), because it has been shown to result from a variety of acoustic changes within a stimulus, such as phoneme, amplitude envelope, and spectrum changes (for a review see Martin et al. (2008)). However, not all acoustic changes result in an N1‐P2 complex. For example, when changes occur in rapid succession, scalp recorded deflections may not occur. Not surprisingly, the amplitude of the response to an acoustic change is smaller in the syllable context (e.g., the response to the vowel in a consonant‐vowel stimulus) than when isolated (e.g., the response to the vowel alone). Smaller amplitudes may be due to both reduced absolute acoustic level change as well as refractory characteristics of populations of neurons. Neural refractoriness, which is distinct from the refractory periods of individual neurons (Umbricht et al. 2004), refers to the time period following an evoked response during which responses to subsequent stimuli are less robust or not present at all (Näätänen and Picton 1987). A common example of refractory effects is seen when decreases in inter‐stimulus interval lead to decreases in N1‐P2 amplitude (although little effect is seen on latency; Davis et al. 1966; Näätänen and Picton 1987). Experiments using more elaborate stimulus paradigms, such as inter‐stimulus intervals varying randomly within each run (Roth et al. 1976) and stimulus trains including deviant tones (Budd et al. 1998), support refractoriness rather than habituation (a process resulting from loss of novelty) as the cause of the response decrement. Refractoriness appears to play a role in the effects of development and age on neural responses, with increased refractory effects documented both in older adults (Papanicolaou et al. 1985; Tremblay et al. 2004) and in young children (Gilley et al. 2005).

In addition to phoneme timing, the presence of background noise complicates the coding task of auditory neurons. It may be that portions of a syllable or word, like softer consonants, are masked by the background noise while other portions, like louder vowels, are still audible. Both phoneme audibility and phoneme timing are important to consider if speech‐in‐noise stimuli are used to evoke neural responses with the intent of clinical applications (e.g., evoked potentials recorded from individuals wearing hearing aids, comparison with behavioral speech perception measures, etc.).

This study investigates the effects of background noise on neural responses to the syllable /sɑ/ and its two constituent phonemes. CAEPs were recorded to phonemes /s/ and /ɑ/ presented both in the context of /sɑ/ and in isolation. Background noise was expected to mask the /ɑ/ and /s/ portions of the syllable differently, given that there are considerable differences between the two phonemes in intensity level, spectral content, and periodicity, which we hypothesized would result in CAEP interaction effects between SNR and acoustic context (within‐syllable vs. isolated).

## Methods

### Participants

Fifteen young, normal‐hearing individuals, ranging in age from 20 to 33 years (mean age = 26.3 years; 7 males and 8 females) participated in this study. All participants were right handed, with pure‐tone thresholds within the normal range (<20 dB HL) from 250 to 8000 Hz and normal tympanometric measures (peak pressure within ± 50 daPa to a 226 Hz tone). All participants were in good general health with no report of significant history of otologic or neurologic disorders. Participants provided written informed consent and the research protocol was submitted to and approved by the Veterans Affairs Portland Health Care System Institutional Review Board.

### Stimuli

Naturally produced speech signals /sɑ/, /s/, and /ɑ/ were presented to the right ear using an ER2 insert earphone. The syllable /sɑ/ was a shortened version (duration reduced from 756 to 450 msec by removing the final 306 msec from the vowel; offset ramped using a 20 msec cosine window) of a female exemplar used previously (Tremblay et al. 2006). The /sɑ/ syllable was then segmented into /s/ and /ɑ/. The /s/ had a duration of 203 msec, followed by 247 msec of silence. The /ɑ/ stimulus was created by zeroing out the /s/ portion of the syllable (from 0 to 203 msec) with the vowel being heard from 203 to 450 msec. Segmentation of /s/ and /ɑ/ was completed at zero‐crossings so as to prevent any audible artifacts. The presentation level of the /sɑ/ signal was 69.1 dBC as measured by a Larson Davis Investigator sound level meter. The /s/ and /ɑ/ stimuli were presented at the same level at which they occurred in the full syllable, preserving natural differences in phoneme intensity (39.3 dBC for /s/, and 71.7 dBC for /ɑ/). The levels of the three tokens were measured using an 8‐sec measurement of a repeated and concatenated version of each token with silent portions removed.

Continuous speech‐spectrum noise used previously (Billings et al. 2011) was added to the background at different levels to create SNRs of 20, 15, 10, 5, 0, and −5 dB relative to the 69 dB presentation level for /sɑ/. This noise has the most energy below 1000 Hz and a steady reduction in energy above 1000 Hz, except for a small peak around 3000 Hz. To reduce time burden and fatigue for participants, only a subset of SNR conditions was used for the /s/ and /ɑ/ stimuli: the /s/ stimulus was presented using only the 20, 10, and 0 dB SNR conditions, while the /ɑ/ stimulus used only the 15, 5, and ‐5 dB SNR conditions. It is important to note that when /s/ and /ɑ/ were presented separately, the nominal SNRs were still defined relative to the overall /sɑ/ rather than to the separate phonemes. The specific SNR conditions used for /s/ and /ɑ/ were motivated by a desire to avoid floor and ceiling effects (i.e., increase the probability of an evoked response while also testing in a range where strong SNR effects might be present). All three stimuli were also presented without added background noise (quiet test environment, QTE) for a total of 15 conditions tested.

### Electrophysiology

Neuroscan^TM^ Stim2 stimulus presentation software was used with the accompanying evoked potential recording system (SynampsRT, Scan 4.5). Each stimulus was presented in a homogeneous train using an inter‐stimulus interval (offset to onset) of 1900 msec. Stimulus type testing order was randomized across subjects, and within each stimulus type, SNR order was randomized across subjects. A single SNR condition was presented in each test block. Subjects were instructed to ignore the stimuli, watch a silent closed‐captioned movie of their choice, and minimize head and body movement. Between 180 and 200 stimulus presentations were recorded for each condition/block. Data collection lasted approximately 2.5 h per participant. Evoked potential activity was recorded using an Electro‐Cap International, Inc. cap which housed 64 tin electrodes. For acquisition, the ground electrode was located on the forehead and the reference electrode was located at vertex. Horizontal and vertical eye movements were monitored with electrodes located inferiorly and at the outer canthi of both eyes. The recording window consisted of a 200 msec prestimulus period and a 1100 msec poststimulus onset period. Evoked responses were analog band‐pass filtered on‐line from 0 to 100 Hz and collected using a sampling rate of 1000 Hz. Eye blinks were removed offline using a spatial filter based on the covariance of blink activity across the scalp. The filter was based on eye blinks that were selected manually from the ongoing electroencephalogram. Following blink removal, trials containing ocular artifacts exceeding ±70 *μ*V were rejected from averaging. Following ocular artifact rejection, the remaining sweeps were averaged and filtered off‐line from 0.1 Hz (high‐pass filter, 12 dB/octave) to 30 Hz (low‐pass filter, 12 dB/octave) and re‐referenced to a common average.

### Data analysis and interpretation

The N1_/s/_ and P2_/s/_ peaks corresponding to fricative onset and the N1_/ɑ/_ and P2_/ɑ/_ peaks corresponding to vowel onset were analyzed from electrode Cz. Peak amplitudes were calculated relative to baseline and peak latencies were calculated relative to 0 msec. For the /sɑ/ and /s/ conditions, 0 msec was at stimulus onset and for the /ɑ/ condition it was 203 msec before stimulus onset due to the silent gap produced by zeroing the /s/. Initially, an automatic peak detection algorithm identified peaks in the following ranges: 140–240 msec for N1_/s/_, 210–310 msec for P2_/s/_, 270–370 msec for N1_/ɑ/_, and 350–450  msec for P2_/ɑ/_; for the poorest SNR tested for each speech token (i.e., 0 or −5 dB), the end of each range was extended by 50 msec. Final peak latency and amplitude values for each peak were then verified by two independent judges. Each judge used temporal electrode inversion, global field power traces, and grand averages to determine peaks for a given condition. Repeated measures analyses of variance (ANOVAs) were completed on amplitude and latency measures of each peak (N1_/s/_, P2_/s/_, N1_/ɑ/_, and P2_/ɑ/_). Two separate two‐factor ANOVAs were completed, including context (i.e., /sɑ/ vs. /s/; /sɑ/ vs. /ɑ/) and SNR as factors. Due to an insufficient number of identifiable peaks for the /s/ condition at 0 dB SNR (peaks were found for only seven or eight of the fifteen individuals tested), only three SNRs were included in the /sɑ/ vs. /s/ ANOVA (2 × 3), whereas the /sɑ/ vs. /ɑ/ ANOVA included all four SNRs tested (2 × 4). Greenhouse–Geisser corrections (Greenhouse and Geisser 1959) were used where an assumption of sphericity was not appropriate.

## Results

**Figure 1:**
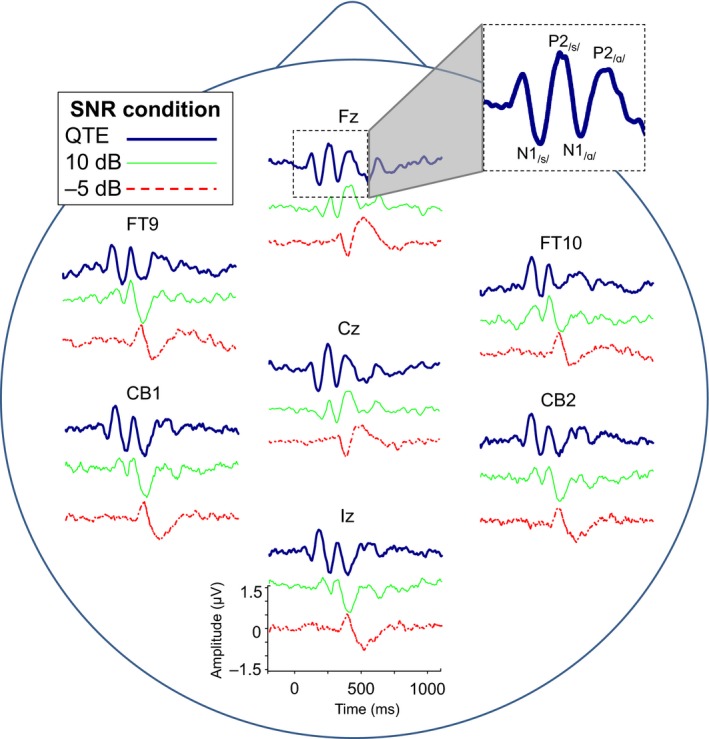
Grand mean evoked potential waveforms to /s/ syllable in quiet, 10 dB SNR, and −5 dB SNR conditions at a subset of electrodes to illustrate similar results across the scalp. A distinct double‐onset response (acoustic change complex) is clear in quiet, with N1_/s/_ and P2_/s/_responses to /s/ and N1_/ɑ/_ and P2_/ɑ/_ to /ɑ/. As SNR worsens, the response to /s/ weakens and disappears while the response to /ɑ/ remains robust.

For all subjects, the syllable /sɑ/ presented without background noise elicited a double‐onset response consisting of both a negative and positive peak corresponding to the onset of the fricative (N1_/s/_ and P2_/s/_, respectively) and an N1_/ɑ/_ and P2_/ɑ/_ corresponding to the onset of the vowel. Figure 1 illustrates the neural responses to the full syllable /sɑ/ for selected electrodes and SNRs. Changes in morphology as a function of SNR are visible across electrodes and similar across the scalp. Two sets of repeated‐measures ANOVA tests were completed, one comparing the response to /s/ (N1_/s/_ and P2_/s/_) when presented in the syllable /sa/ or in isolation, and another comparing the response to /a/ (N1_/ɑ/_ and P2_/ɑ/_) when presented in the syllable /sɑ/ or in isolation.

### Effects of SNR

**Figure 2:**
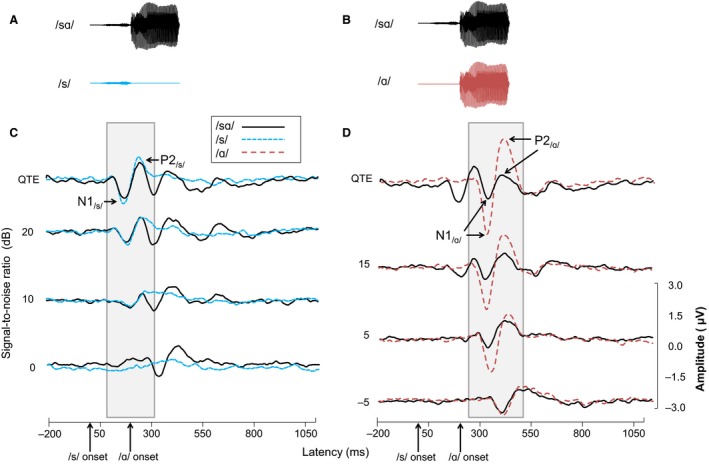
Stimulus time waveforms and grand mean evoked potential waveforms recorded from electrode Cz. (A), (B) Stimulus waveforms for /sɑ/ and /s/ tokens and /sɑ/ and /ɑ/ tokens are shown with shaded boxes indicating the peaks of interest for each comparison. (C) Grand mean CAEP waveforms to the /s/ as a function of SNR are similar across syllable context, whether in the syllable or isolation. (D) Grand mean CAEP waveforms to /ɑ/ as a function of SNR differ depending on whether the vowel is presented in syllable context or isolation. At good SNRs, responses to /ɑ/ have a greater amplitude than responses to the vowel in /sɑ/; this effect of syllable context disappears as SNR decreases. All SNR values reference the RMS signal levels of the full syllable rather than the signal levels of the individual phonemes.

Each isolated phoneme (/s/ and /ɑ/) elicited a single N1‐P2 complex with latencies consistent with the onset time of the given phoneme (Fig. 2C and D). Corresponding acoustic waveforms are also shown (Fig. 2A and B). Systematic effects of SNR are evident, with N1 and P2 latencies generally increasing and amplitudes generally decreasing as SNR worsens. Repeated‐measures ANOVAs revealed main effects of SNR on latencies and amplitudes for all peaks (N1_/s/_ latency: *F*_(1.2,11.2)_ = 11.5, *P* = 0.004; P2_/s/_ latency: *F*_(2,18)_ = 16.8, *P* < 0.001; N1_/s/_ amplitude: *F*_(1.1,10.1)_ = 9.2, *P* = 0.011; P2_/s/_ amplitude: *F*_(2,18)_ = 7.6, *P* = 0.004; N1_/ɑ/_ latency: *F*_(3,36)_ = 202.1, *P* < 0.001; P2_/ɑ/_ latency: *F*_(3,27)_ = 75.5, *P* < 0.001; N1_/ɑ/_ amplitude: *F*_(3,36)_ = 15.6, *P* < 0.001; P2_/ɑ/_ amplitude: *F*_(3,27)_ = 10.0, *P *< 0.001).

**Figure 3:**
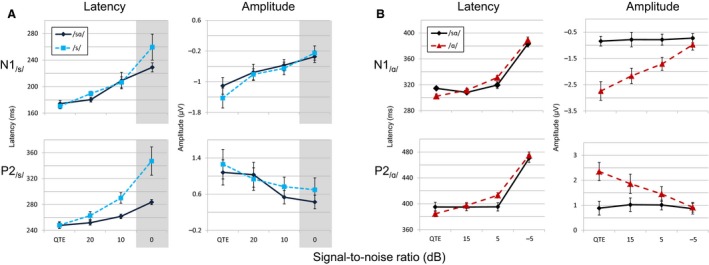
Latency and amplitude growth functions with changes in SNR. (A) Mean evoked response N1_/s/_ and P2_/s/_ latencies and amplitudes for the consonant in /sɑ/ and /s/ tokens across SNR conditions are shown. The 0 dB SNR condition (shown in gray) was not included in the /sɑ/ and /s/ ANOVA due to an insufficient number of identifiable peaks. (B) Mean N1_/ɑ/_ and P2_/ɑ/_ latencies and amplitudes for the vowel in /sɑ/ and /ɑ/ tokens across SNR conditions demonstrate a context‐by‐SNR interaction effect on vowel response amplitudes. The interaction probably results from a combination of stimulus level and refractory effects. Error bars represent standard error of the mean.

CAEP response amplitude to the fricative /s/ in both contexts decreased dramatically as SNR decreased, such that the response disappeared at the poorest SNRs. Mean N1_/s/_ and P2_/s/_ peaks for the isolated fricative and the fricative in the context of /sɑ/ are seen in Figure 3A, along with N1_/ɑ/_ and P2_/ɑ/_ means for the isolated vowel and the vowel in context (Fig. 3B). As SNR decreased, the amplitudes of the N1_/s/_ and P2_/s/_ to the /s/ in /sɑ/ became smaller, while N1_/ɑ/_ and P2_/ɑ/_ to the /ɑ/ in /sɑ/ remained relatively stable.

### Effects of context

Overall, responses to /s/ were similar regardless of context as can be seen in Figure 3A. Shaded areas represent the incomplete dataset for the poorest SNR condition that resulted in the 2 × 3 ANOVA. Repeated‐measures ANOVA tests revealed no significant effect of context (/s/vs. /sɑ/) on P2_/s/_ latency or amplitude. As with the /s/ in the full syllable, responses to the /s/ in isolation were nearly absent at the poorest SNRs (see Fig. 2). Neural response amplitudes to /ɑ/, however, were highly dependent on context (Fig. 3B). At better SNRs, responses to /ɑ/ in isolation had larger amplitudes than responses to /ɑ/ in syllabic context. The effect of context (/ɑ/ vs. /sɑ/) on N1_/ɑ/_ and P2_/ɑ/_ amplitudes was significant (N1_/ɑ/_: *F*_(1,12)_ = 50.4, *P* < 0.001; P2_/ɑ/_: *F*_(1,9) _= 24.5, *P* = 0.001). Unlike the /s/ tokens, neural responses to /ɑ/ in both contexts were present even at the poorest SNRs.

### SNR‐by‐context interaction

While peak response amplitudes to the isolated /ɑ/ decreased with SNR, amplitudes in response to /ɑ/ in syllable context were much more stable across SNRs. This effect of context decreased with decreasing SNR, such that at the poorest SNR, N1_/ɑ/_ and P2_/ɑ/_ amplitudes became comparable across contexts. Repeated‐measure ANOVAs indicated a significant interaction of SNR by context (/ɑ/ vs. /sɑ/) for amplitudes for both peaks (N1_/ɑ/_: *F*_(3,36)_ = 11.6, *P* < 0.001; P2_/ɑ/_: *F*_(3,27)_ = 7.7, *P* = 0.001) as well as for N1_/ɑ/_ latency (*F*_(3,36)_ = 4.0, *P* = 0.016). In contrast, for the /s/ phoneme, only the P2_/s/_ latency showed a significant SNR‐by‐context interaction (*F*_(2,18) _= 6.0, *P* = 0.01).

## Discussion

As anticipated, CAEP responses generally became weaker with increasing background noise; as SNR decreased, peak latencies increased and amplitudes decreased. While neural responses to /s/ at the poorest SNRs were absent or very weak, responses to /ɑ/ were present at all SNRs. These results are consistent with stimulus audibility relative to the background noise. The intensity of the /s/ was 30 dB lower than the /ɑ/, causing it to be completely, or near‐completely, masked at the poorest SNR while the higher intensity of the /ɑ/ allowed for detection and clear neural responses even at the poorest SNR. Differences in periodicity between the phonemes may also have contributed to this effect. Vowels (such as the /ɑ/ phoneme used here) are periodic, while fricatives (such as the /s/) are not. Thus /ɑ/ was likely more salient against the aperiodic background noise than was /s/. This is consistent with previous findings that the ACC can be evoked by changes in periodicity alone (e.g., Martin and Boothroyd 1999). Spectral differences between the two phonemes are also important to consider. The /s/ had relatively greater high‐frequency energy (energy focused between 2500 and 8000 Hz) than the background noise, making it more distinct from the noise spectrum than the /ɑ/, which was primarily low‐frequency focused (energy dropping off above 1600 Hz). However, it should be noted that relative levels of each phoneme were quite different (39.3 dBC for /s/, and 71.7 dBC for /ɑ/) and likely had a larger effect than the spectral differences between phonemes.

In quiet, evoked responses to the /ɑ/ in isolation showed larger amplitudes than responses to the /ɑ/ in syllable context, while context had little overall effect on responses to /s/. Ostroff et al. (1998) also demonstrated that the neural response to a vowel, when preceded by a consonant, is smaller than when the vowel is presented in isolation. However, the interaction between context and SNR (i.e., a strong SNR effect on N1_/ɑ/_ amplitude in isolation but not in syllable context) is novel to this study. Interestingly, Ganapathy and Manjula (2016) recently showed an effect of SNR on peak‐to‐peak response amplitude to the /ɑ/ using a /sɑ/ stimulus, although the amplitude effect was only found at the most favorable SNRs. These mixed results may indicate that multiple effects are at work. Refractoriness and relative level changes between phonemes are two potential explanations for such different effects of context on the two phonemes.

Because of the temporal proximity of the fricative to the vowel, at vowel onset many neurons may be in recovery from their response to the fricative and unable to respond to the vowel. Thus, the response to /ɑ/ in syllable context is lower in amplitude than the response to the isolated /ɑ/. Level differences may also be at play; the difference in stimulus intensity level between the pre‐stimulus period and the onset of the isolated /ɑ/ is greater than the difference in level between the /s/ and /ɑ/ in syllable context. The larger stimulus amplitude increase when the /ɑ/ is in isolation could explain the larger neural onset response for the /ɑ/ in isolation compared to the /ɑ/ in syllable context.

Amplitudes and latencies of the response to /ɑ/ in syllable context or in isolation probably result from a combination of level changes (i.e., level differences between the /s/, /ɑ/, and background noise) and refractory effects (refractory periods following the /s/ whose durations encompass the following /ɑ/ onset). Presumably, the largest refractory effects would be present in the quiet condition when the /s/ is completely audible and there is some overlap between the neural activity to the /s/ and the neural activity to the /ɑ/. However, as noise level is increased, more and more of the /s/ would be masked, resulting in less overlap, and therefore, smaller refractory effects. Noise level effects, on the other hand, would be smallest in the quiet condition and gradually increase (weakening the evoked response) as noise level increases. Therefore, a tradeoff between refractory effects and level effects may be occurring such that amplitude of the N1_/ɑ/_ remains unchanged with SNR. Certainly, it is clear that for an SNR of ‐5 dB, the level effects would dominate, and for the quiet condition, the refractory effects would dominate. Interestingly, latency changes essentially do not occur until the poorest SNR condition, which is consistent with the idea that refractory effects dominate at more favorable SNRs, since these have been shown to impact amplitude without affecting latency (Davis et al. 1966). It should be noted that the potential refractory effects seen here may improve coding of complex sounds. For example, Berry and Meister (1998) demonstrated that refractoriness may actually improve neuronal reliability in individual neurons rather than limit the performance of a neuron when considered over a period of time. That is, refractory periods may limit the firing of a neuron at a given point in time, but if considered over time, the firing may become more regular, improving the temporal precision of subsequent spikes to a stimulus. With electroencephalography, although postsynaptic potentials of populations of neurons drive the response, the same principles may apply.

There was an unexpected significant interaction of SNR and context on P2_/s/_ latency. This may be due to overlapping P1‐N1‐P2 responses to the consonant and vowel and the difficulty differentiating the P2_/s/_ from the P1 elicited by the /ɑ/ onset when calling peaks in the waveforms to /sɑ/. Peaks elicited only by the vowel onset and not by the fricative (i.e., solely P1_/ɑ/_ and not P2_/s/_) may have been erroneously identified as P2_/s/_, which would have inflated the number of early‐latency P2_/s/_ peaks and led to the apparent significant interaction.

Robust effects of SNR were found for the fricative /s/ both in isolation and syllable context, consistent with previous findings. Interestingly, neural response amplitude to the vowel in syllable context remained relatively stable across SNRs, probably resulting from a tradeoff between faithful coding of stimulus acoustics (e.g., level, spectrum, periodicity) and the effects of neural refractoriness. Results suggest that neural coding of complex speech in background noise is not uniformly affected by SNR; instead, some responses appear to remain relatively robust where others are eliminated. The relative contributions of these effects will vary depending on the characteristics of the stimulus that is used. For example, two subsequent phonemes with similar intensity and spectral properties would be masked similarly and refractory effects may be greater because of the greater overlap between neural populations activated by each phoneme, likely resulting in a greatly reduced acoustic change response. Evoking the acoustic change complex in background noise using additional complex speech signals, in conjunction with behavioral testing, is needed to further the understanding of speech‐in‐noise perception, with the future goal of improving diagnosis and treatment for patients who struggle with speech perception in noise.

## Conflict of Interest

None of the authors has potential conflicts of interest to be disclosed.
